# The development of an intervention for diabetes prevention among people with impaired glucose regulation: feasibility and acceptability of an intervention component

**DOI:** 10.1186/s40814-019-0435-4

**Published:** 2019-04-04

**Authors:** Josie M. M. Evans, Linda Irvine, Jenni Connelly, Dawn M. Cameron

**Affiliations:** 1 Faculty of Health Sciences and Sport, University of Stirling, Stirling, FK9 4LA UK; 2 School of Nursing and Health Sciences, University of Dundee, Dundee, UK

## Abstract

**Background:**

As part of the design process of a low-cost minimal-contact diabetes prevention intervention, we issued a blood glucose meter to people with impaired glucose regulation (who are at high risk of type 2 diabetes). We conducted a feasibility study to assess the acceptability of this intervention component and whether and how recipients engaged with it.

**Methods:**

A blood glucose meter was given to 19 people identified through primary care, who were asked to use the meter in an exploratory way during a 4-week trial period, to try to understand the effect of different foods on the body. They were advised that they could test as often or as little as they liked and were also asked to keep a food/exercise diary for at least 1 week. They were interviewed about their experiences afterwards.

**Results:**

There was a high level of engagement with testing, with the total number of tests recorded ranging from 11 to 114 (median 74) among 18 participants. Fifteen participants tested almost every day during the 4-week period. The cognitive engagement was more limited. All participants commented on their own results, and most were able to relate high or low results to foods eaten and exercise taken, usually in response to prompting. However, there was limited thought or understanding beyond this in terms of longer-term patterns of diet and exercise, and testing was routine rather than experimental. Some participants were confused by conflicting or unexpected results. A few minor problems were reported by participants, such as soreness, inconvenience, and difficulty in getting blood, but never enough to discontinue testing. Several participants stated that the meter was a useful aid as a reminder that they were at high risk of diabetes and served as a prompt that needed to make and/or maintain behaviour changes.

**Conclusions:**

The study suggests that blood glucose monitoring is acceptable to people with impaired glucose regulation and that they would engage with it as part of an intervention to improve their diet. The study has also uncovered potential mechanisms of action for behaviour change.

## Background

The incidence and prevalence of type 2 diabetes and its complications are increasing rapidly and presenting huge clinical and financial challenges to the NHS. The prevalence of impaired glucose regulation (IGR) (sometimes referred to as pre-diabetes) in the general population is as high as 15% in Europe [1]. People with IGR are at high risk of developing type 2 diabetes. However, there is sound empirical evidence that lifestyle changes relating to diet and physical activity can reduce the risk of progression to type 2 diabetes among high-risk individuals [2].

Dietary behaviour remains one of the most difficult ‘lifestyle’ behaviours to change. Two of nine RCTs in a systematic review of lifestyle interventions for people at high risk for type 2 diabetes (which all addressed diet, exercise and at least one other component) showed only small improvements in dietary outcomes [3]. Although simply giving people information about being healthy is rarely sufficient to change their behaviours, there is a need for at least basic knowledge and understanding, particularly for a complex behaviour such as eating a healthy diet. In particular, there is substantial dietary-related knowledge that needs to be assimilated, and an understanding of the effects of different foods and drink on the body. In people with newly diagnosed type 2 diabetes in the UK, lack of knowledge and understanding was identified as a key barrier to dietary self-management, even after structured diabetes education [4]. This is likely to be even more pronounced among people at high risk of diabetes who do not have access to such education. The challenge is to ensure that people with IGR have the knowledge that they need to make effective dietary changes.

Experiential learning is the process of learning through experience; more specifically defined as ‘learning through reflection on doing’ [5]. Introducing an element of experiential learning to people known to be at high risk of type 2 diabetes and for whom dietary changes are important, might result in a better understanding of the eight key messages of healthy eating, as advocated by NHS Public Health England [6].

We are therefore designing a low-cost minimal-contact educational intervention for people with IGR that includes experiential learning through the use of a blood glucose meter. This intervention component was suggested by a layperson with diabetes. A key area of uncertainty was whether this component would be acceptable to potential recipients and whether and how they would use it. The aim of this feasibility study was therefore to assess the acceptability of the use of a blood glucose meter in this population, to determine whether people with IGR would engage with it and to explore their views and experiences of using it. The study also informed potential mechanisms of action of the intervention component.

## Methods

Recruitment for this study took place in primary care. A number of general practices within two health boards in Scotland, UK, were sent information. Seven practices agreed to assist with recruitment, and a representative from the NRS Primary Care Network visited each practice to access electronic records to identify English-speaking adults for whom there was a record of a diagnosis of IGR in the previous year, as a result of a blood test. General practitioners were asked to check that potential participants had already been informed of their IGR diagnosis, and that there was no other specific reason to not include them in the study (e.g. cognitive impairment). A letter of invitation was then sent to them, together with a participant information leaflet, and a detailed dietary guide (the Eatwell guide [6]).

The participants were asked to return an opt-in form with their contact details if they were interested in taking part in the study, at which point a first visit was arranged which took place either in their home (*n* = 18) or at the University of Stirling (*n* = 1). This was undertaken by LI, a researcher employed on the study with previous experience of qualitative research, but no previous contact with any participant. Written consent was taken, then participants were asked questions about their understanding of their IGR diagnosis and shown how to use the meter according to NHS protocol. It was suggested that they use the meter in an exploratory way during a 4-week trial period, to try to understand the effect of different foods on the body, but that they could test as much or as little as they liked. For example, we suggested that they might wish to consider their results in relation to whether they had eaten healthily that day or not. They were also asked to keep a food/exercise diary for at least 1 week (but up to 4 weeks if convenient to them), also noting down their readings. Although the meter did have more advanced functionalities (e.g. storing readings, charting results), these functionalities were not expressly shown to participants. Dietary pre-education was minimal and simply involved revisiting the dietary guide that participants had received prior to the interview and asking whether any clarification was needed.

After the 4-week period had elapsed, they took part in a semi-structured interview to explore their views and experiences of using the meter. The semi-structured format of the interview ensured that topics of interest were covered while allowing participants the freedom to discuss any issues not covered in the guide. The topics covered were the acceptability of the intervention, how and when participants tested, whether they found testing useful, a discussion of their diary entries, and whether they think testing helped their understanding of the key dietary messages.

All interviews were audio-recorded with the participant’s permission and transcribed verbatim (but not returned to the participants). Data were managed using NVivo and analysed using a framework method, which is often advocated for projects with multi-disciplinary research teams [7]. Within this method, data are coded, indexed and charted systematically. Two members of the research team read and coded all transcribed interviews. For this paper, all data that had been coded as relating to the specific topics were manually retrieved and analysed, and discussed within the research team.

Approval to conduct the study was obtained from a National Health Service (NHS) Research Ethics Committee.

## Results

A total of 81 people from 7 practices were initially identified as eligible for the study, to whom 62 letters of invitation were sent. Twenty people returned an opt-in form, all of whom subsequently agreed to take part, but only 19 were visited (it was not possible to identify a mutually convenient time to visit one participant). Recruitment details are presented in Table 1. There were 14 men and 5 women; age ranged from 45 to 76 years.

**Table 1:** Details of recruitment of general practices and participants

General practice	Eligible	Invitation letter sent	Opted in	Recruited	Age and sex
Rural	8	6	0	0	–
Small, relatively affluent town	9	8	4	4	F, 65–69 M, 75–79 M, 65–69 M, 60–64
City, relatively deprived area	21	20	5	5	F, 75–79 M, 65–69 M, 55–59 M, 55–59 M, 60–64
Small city, relatively affluent	12	9	4	3	F, 65–69 M, 70–74 M, 70–74
City, relatively deprived area	14	5	3	3	F, 55–59 M, 65–69 M, 45–49
City, relatively deprived area	17	14	4	4	F, 55–59 M, 60–64 M, 60–64 M, 70–74

One participant was unable to use the glucose meter at the first visit and was withdrawn from the study. There was a high level of engagement with the testing itself among the 18 remaining participants, with the total number of tests carried out and recorded ranging from 11 to 114 (median 74). Fifteen participants tested at least once almost every day during the 4-week period. The exceptions were a female participant who tested for only 2 weeks before going on holiday; a male participant who tested for 4 days only as he then went onto a fluid-only pre-operative diet; and a female participant who tested for 5 days only as she did not find testing useful.

### Acceptability

There were no participants who questioned the acceptability of giving meters to other people like themselves. A few specific problems were mentioned, such as soreness, inconvenience and difficulty in getting blood, and but not enough to discontinue testing. Some participants asked to keep the meter. Two participants had already bought extra testing strips for themselves, although the cost of the strips (up to £15 for 50) was mentioned as a potential barrier to this: ‘they’re quite expensive so it kind of puts you off a wee bit’ (female, aged 66).

### Engagement with the meter

Cognitive engagement with the meter was fairly limited. All participants volunteered comments about their own results, and most tried to relate high or low results to specific foods eaten and exercise taken, usually in response to prompting. While many mentioned the effects of sweetened food, there was little consideration of other food groups, and very little exploration or experimentation with testing; for example, after different foods and drinks, or at varying times. In consequence, there was limited thought (or indeed understanding) about how participants might make longer-term changes to their dietary and exercise behaviours in order to avoid extreme readings. Therefore, the use of the meter did not appear to particularly enhance knowledge and understanding. Another reason for this was that testing was simply a routine activity for some participants. For participants who were more interested in analysing their own results and had agreed to the study expressly to develop their knowledge and understanding, testing seemed to generate questioning, confusion and concerns, particularly when results appeared to fluctuate randomly and widely, as indicated by the following quotes:

‘And I said, “I’m on this trial thing, could you explain why it’s going up and down?”. I said “Because I’m getting all the different readings when I’m being good to when I’m doing badly’”. (male, aged 59)

‘I have no idea why it moved around the way it did’ (male, aged 75)

Despite this lack of understanding around specific dietary effects, several participants still believed that the meter was a useful aid to make them more aware or *‘*more conscious’ (male, aged 59), and act as a reminder or prompt that they were at high risk of diabetes and needed to make and/or maintain behaviour changes.

‘so, yeah, you do think more about it … .on a daily basis as well’. (male, aged 70)

This effect subsided relatively quickly for some participants, but not necessarily for all.

‘And what I would say is that since I have stopped using it, I managed to put all these thoughts to the back of my mind. So if you like, it made the possibility of being diabetic a little more prominent in my mind than it would’ve done’. (male, aged 75)

### Behaviour change

Some participants reported that they had begun to change their behaviour, again despite not necessarily having a fully developed understanding of why this might improve glycaemic control.

‘I’m starting to walk more’. (male, aged 65)

‘I’m eating much better now’. (male, aged 63)

‘I now look at things in supermarkets and say oooh oh no, and put it back down again, it may be too much sugar, so yeah I’m more aware’ (male, aged 70)

**Fig. 1:**
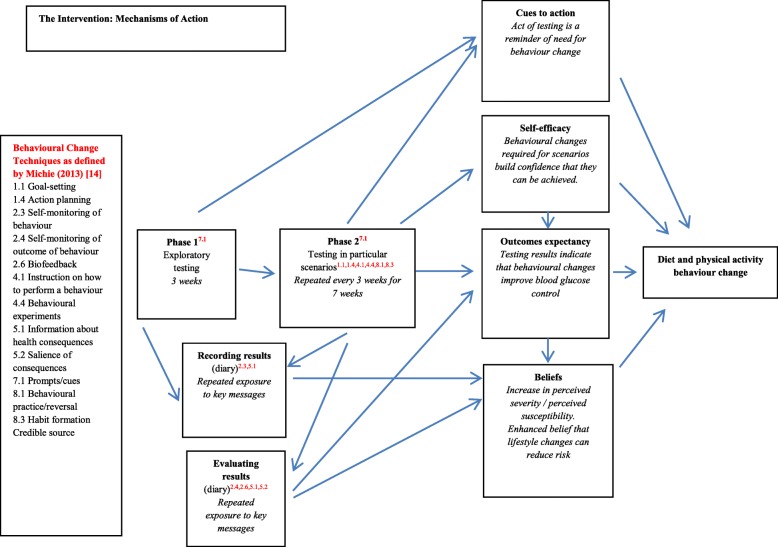
Mechanisms of action of the finalised educational intervention

While many of these changes were relatively minor and short-lived, the results suggest that the meter may have potential as a prompt or a reminder for behavioural change. The hypothesised mechanisms of action of the proposed intervention that were refined in response to these results are summarised in Fig. 1.

## Discussion

The results from this feasibility study provide strong evidence for the acceptability of the blood glucose meter as a component of an intervention. For example, there was an opt-in proportion of 32%, which is encouraging given that opt-in strategies are known to generate lower response rates in research studies [8]. All participants who opted in were then initially recruited and most tested frequently. However, two of the three participants who did very little testing had practical reasons for this. The third was a retired health care professional who felt that testing was unlikely to be educational for her. Only one participant could not use the meter, as he was unable to obtain a blood sample.

Cognitive engagement with blood glucose monitoring was minimal, as has also been noted among people with type 2 diabetes [9]. Most participants tested routinely with limited reflection. This may be because we provided very limited guidance as to how and when they might test, but allowed participants the freedom to experiment as they wished. However, we now suggest that more directed guidance be given to them, with suggestions and options as to when, how and under what conditions experimental testing might be carried out.

An important finding from this feasibility study was that some participants had started to think about or had even made some dietary and physical activity behavioural changes. This seemed to be partially in response to having the blood glucose meter as a prompt or a reminder that they had a condition, and was apparent even in the absence of a more developed understanding of the effects of diet and exercise on the body. It is known that IGR is under-treated in primary care [10], and while there has been very little research around what people themselves understand by having the condition, a study in the USA demonstrated low diabetes identity perceptions among a group of people at high risk of diabetes [11]. Women known to be at high risk of diabetes by virtue of previously having had gestational diabetes show similar lack of awareness/concern [12]. It may be that the issue and continued presence of a blood glucose meter to people with IGR may operate as a repeated cue to action for behaviour change (as defined in the Health Belief Model [13]). Another possibility is that it might serve as a prompt to move people between stages of the trans-theoretical model of behaviour change (e.g. pre-contemplation to contemplation, or contemplation to action) [14].

The opt-in group of participants may represent people who are more interested in health and more motivated to make changes, than the general population of people with IGR. The sample was relatively large for the qualitative element of the study; and although not selected to be statistically representative of people with IGR, it was a heterogeneous sample. The five GP practices were fairly diverse in terms of population served, with the participants coming from a range of different socio-economic backgrounds. There was a disproportionately high number of male participants in our study, which may have affected the findings. Despite these limitations, the study strongly suggests that the intervention component would be acceptable to the intended recipient population and that they would engage with it. However, it also indicates that participants may require more detailed guidance in order to maximise the potential of the blood glucose meter use for enhancing dietary education.

The study has provided insight into potential mechanisms of action of the intervention component, and we have refined the final intervention accordingly. The proposed intervention now takes the form of a short initial period of exploratory testing, followed by a second longer phase of less frequent testing, where recipients are guided as to timing and context, with example scenarios of how and when they might test (for example, after exercise or following a large meal). They are encouraged to record their results in a diary that exposes them to educational prompts and messages relating to diabetes risk, and suggestions for behaviour change. The proposed mechanisms of action are summarised in Fig. 1. If intervention recipients engage with the testing, we suggest that illness beliefs would be addressed by repeated exposure to key messages relating to risk. Suggested dietary and physical activity experimentation in testing scenarios would build confidence this small behaviour changes can be made, and that they have an effect on blood glucose control (outcomes expectancy). The testing process would operate as a cue to action for behaviour change. The Health Belief Model hypothesises that all these constructs are related to sustained behaviour change [13]. Figure 1 also lists the behaviour change techniques incorporated into the intervention [15]. The next step in our research programme is to pilot the finalised intervention.

## References

[1] Eades CE, France E, Evans JMM (2016). Prevalence of impaired glucose regulation in Europe: a meta-analysis. Eur J Pub Health.

[2] Yoon U, Kwok LL, Magkidis A (2013). Efficacy of lifestyle interventions in reducing diabetes incidence in patients with impaired glucose tolerance: a systematic review of randomized controlled trials. Metabolism.

[3] Schellenberg ES, Dryden DM, Vandermeer B, Ha C, Korownyk C (2013). Lifestyle interventions for patients with and at risk for type 2 diabetes: a systematic review and meta-analysis. Ann Intern Med.

[4] Booth AO, Lowis C, Dean M, Hunter SJ, McKinley MC (2013). Diet and physical activity in the self-management of type 2 diabetes: barriers and facilitators identified by patients and health professionals. Prim Health Care Res Dev.

[5] Kolb DA (1984). Experiential learning: experience as the source of learning and development.

[6] Public Health England. The Eatwell guide. https://www.gov.uk/government/publications/the-eatwell-guide. Accessed 4/7/2018.

[7] Gale NK, Heath G, Cameron E, Rashid S, Redwood S (2013). Using the framework method for the analysis of qualitative data in multi-disciplinary health research. BMC Med Res Methodol.

[8] Junghans C, Feder G, Hemingway H, Timmis A, Jones M (2005). Recruiting patients to medical research: double blind randomised trial of “opt-in” versus “opt-out” strategies. Br Med J.

[9] Cameron DM, Harris F, Evans JMM (2018). Self-monitoring of blood glucose in insulin-treated diabetes: a multi case study. BMJ Open Diabetes Res Care.

[10] Gillett M, Royle P, Snaith A, Scotland G, Poobalan A, Imamura M, Black C, Boroujerdi M, Jick S, Wyness L, McNamee P, Brennan A, Waugh N. Non-pharmacological interventions to reduce the risk of diabetes in people with impaired glucose regulation: a systematic review and economic evaluation. Health Technol Assess. 2012;16(33). 10.3310/hta16330 PMC4780928 22935084

[11] Strauss SM, Rosedale MT, Kaur N (2015). Illness perceptions among adults at risk for diabetes. Diabetes Educ.

[12] Eades CE, France E, Evans JMM (2018). Postnatal experiences, knowledge and perceptions of women with gestational diabetes. Diabet Med.

[13] Hochbaum GM. Public participation in medical screening programs: a sociopsychological study: United States Public Health Service. Public Health Publication No. 52; 1958.

[14] Prochaska JO, Velicer WF (1997). The transtheoretical model of behaviour change. Am J Health Promot.

[15] Michie S, Richardson M, Johnston M, Abraham C, Francis J, Hardeman W, Eccles MP, Cane J, Wood CE. The behavior change technique taxonomy (v1) of 93 hierarchically clustered techniques: building an international consensus for the reporting of behavior change interventions. Ann Behav Med. 2013:81–95. 10.1007/s12160-013-9486-6 23512568

